# Evaluation of salivary transferrin in patients with oral squamous cell carcinoma

**DOI:** 10.1002/cre2.809

**Published:** 2023-11-14

**Authors:** Fatemeh Tavakoli, Mohammad Ali Ghavimi, Vahid Fakhrzadeh, Dorna Abdolzadeh, Aylar Afshari, Hosein Eslami

**Affiliations:** ^1^ Department of Oral and Maxillofacial Medicine, School of Dentistry Shiraz University of Medical Sciences Shiraz Iran; ^2^ Department, of Oral and Maxillofacial Surgery, School of Dentistry Tabriz University of Medical Sciences Tabriz Iran; ^3^ Department of Prosthodontics, School of Dentistry Tabriz University of Medical Sciences Tabriz Iran; ^4^ School of Dentistry Tabriz University of Medical Sciences Tabriz Iran; ^5^ School of Dentistry Shiraz University of Medical Sciences Shiraz Iran; ^6^ Department of Oral and Maxillofacial Medicine, School of Dentistry Tabriz University of Medical Sciences Tabriz Iran

**Keywords:** oral squamous cell carcinoma, salivary biomarkers, transferrin

## Abstract

**Objectives:**

About 94% of oral cancers are squamous cell carcinomas (OSCCs). Its occurrence is age‐related due to some factors. Salivary biomarkers have good susceptibility to OSCC's early diagnosis. Moreover, since the clinical diagnosis of advanced stages of OSCC is feasible, its prognosis is very poor.

**Material and Methods:**

According to inclusion and exclusion criteria, 40 OSCC patients and 40 healthy people were selected, and 5 mL of saliva were prepared from each person. The quantity of saline transferrin was computed. After that, the data were analyzed.

**Results:**

Our study results demonstrated that the mean and standard deviation of the salivary transferrin in the control group were 1.234 mL and 0.374, respectively, and in the case group, it was equal to 2.512 mL for the mean and 0.463 for the standard deviation. There was a statistically substantial difference between the mean of the salivary transferrin variable in the two study groups.

**Conclusion:**

In conclusion, the mean concentration of salivary transferrin in the case group was higher than in the control group.

## 1 INTRODUCTION

Oral cancer is one of the major health problems in the world due to its high incidence rate and low survival rate (Eslami et al., 2022; Tuominen & Rautava, 2021). The number of cases of oral cancer is especially high in men (Abati et al., 2020). The number of cases of oral cancer is especially high, making it the eighth most prevalent cancer in the world. About 94% of all oral malignancies are squamous cell carcinoma (SCC) (Narayanasamy Rajavelu et al., 2020). The most common site of occurrence is the tongue area (Mortazavi et al., 2019). The incidence of oral cancer is age‐dependent, and this can reflect the time required to accumulate genetic changes, the accumulation of exposure to initiating and promoting factors, and the decrease in immunity due to aging (D'Souza & Kumar, 2020). The survival rate of 5 years for people with oral carcinoma is below 50%. Despite advances in radiotherapy, surgery, and chemotherapy, the survival rate has not improved (Uma Maheswari et al., 2020). Although with early diagnosis of mouth lesions and proper following treatment, it is possible that the survival rate increases (Stepan et al., 2023).

SCC is a multifactorial disease. No causal factors were identified and agreed upon (Piemonte et al., 2022). Although, some external and internal factors were suggested (Chen et al., 2018; Johnson et al., 2020). Oral SCC (OSCC) may be flat or raised, wounded or unwounded, slightly palpable or indurated. Lymphatic spread of oral carcinoma is possible. Cancer‐related lymph nodes are gradually enlarged and have a hard texture. Lymph nodes are not tender with touch unless there is a secondary infection or an inflammatory response from the biopsy (De Cicco et al., 2021; Maia et al., 2018; Mountcastle et al., 2020).

The gold standard for diagnosing oral cancer is surgical biopsy. Supplementary tools such as toluidine blue vital staining and autofluorescence imaging have been developed and studied to aid clinicians in the diagnostic pathway (Abati et al., 2020).

The identification of tumor biomarkers is the first step necessary to develop a system for the early diagnosis of oral lesions and malignancies. Clinical tests and biopsies are standard methods for the identification of oral ulcers. However, oral cancers are often only discovered in the late stages, and occasionally, a biopsy sample may not be drawn up from the part in which the dysplasia is present (Buzalaf et al., 2020; Kwong et al., 2021). Other screening methods, such as imaging, are available, but they are time‐consuming and costly. Therefore, Researchers are seeking simpler ways to make an early diagnosis (Al‐Garadi et al., 2020).

In the past few years, numerous studies have been performed on biomarkers of blood and saliva for cancer diagnosis. The results of some of these studies have shown that saliva is filled with DNA, RNA, exosomes, and so forth, which contain proteins that come from cancer cells. Therefore, researchers have suggested that saliva can be used for early diagnosis of diseases (Kaya et al., 2022; Tenchov et al., 2022). Similar to serum, saliva contains various enzymes, hormones, antibodies, antimicrobial compounds, and effective growth factors. As a diagnostic fluid, saliva has significant advantages over serum. Such as being noninvasive, not requiring complex training for people to collect it, and the possibility of being collected with simple supplies and equipment. There is no limit in terms of volume for collection, and the procedure costs are low. Saliva analysis is cost‐effective for screening larger populations; therefore, the use of saliva and salivary biomarkers can be a useful and practical step in the diagnosis (Chadha et al., 2022; Huang et al., 2023; Roi et al., 2020).

Salivary biomarkers such as transferrin are suitable for early diagnosis of OSCC (Riccardi et al., 2022). Transferrin is a protein that carries iron in the blood (Shen et al., 2018). It is made in the liver in the form of inactive protein apo transferrin, and it activates when it bonds with ferric ions (Farhadian et al., 2022; Kawabata, 2019). Transferrin is a protein that transports iron and can be measured directly (Kawabata, 2019). It is essential for the growth of fast‐growing cells and is involved in iron‐requiring metabolic processes such as DNA synthesis, electron transfer, metabolic signaling pathway, and cell proliferation and survival (Andrade et al., 2020). Furthermore, overexpression of transferrin receptor has been reported in various cancers such as lung cancer, glioma, colon cancer, pancreatic cancer, and breast cancer (Shen et al., 2018).

Recent studies have shown that transferrin improves the transfection of cationic liposome/DNA complex into OSCC cells (Halib et al., 2019). In a study, it was shown that salivary transferrin has a suitable sensitivity and specificity for the early diagnosis of OSCC (Buzalaf et al., 2020). However, being limited to a few studies is not enough, and accurate conclusions require more studies.

Taking into account that since the clinical diagnosis of OSCC is generally feasible in the advanced stages of the disease and in spite of complex and advanced treatments, its prognosis is very poor and early detection of this lesion is very beneficial, the present study was designed and implemented with the aim of evaluating the amount of salivary transferrin in patients with OSCC.

## 2 MATERIALS AND METHODS

### 2.1 Study design

In this study, the target population was people with OSCC. The sampling method was simple random sampling. The sample size was based on the study of Abati et al. (2020). Considering *α* = .05, the power of the study was 80%, the difference between three units in the amount of salivary transferrin of sick and healthy subjects was seen (17 people). For more certainty, with an increase of 20%, the number of 40 people with oral SCC and the number of 40 healthy people were selected based on the inclusion and exclusion criteria. After obtaining informed consent, they entered the study. The protocol of the clinical trial was conducted according to the ethical principles of Helsinki (version 2002) and was approved by the ethics committee of Tabriz University of Medical Sciences (IR.TBZMED.REC.1397.814). This study was conducted at faculty of dentistry of Tabriz University of Medical Sciences.

### 2.2 Inclusion and exclusion criteria of study groups

The inclusion criteria for the group with OSCC: people aged 20–90 years suffering from head and neck cancer which was confirmed with biopsy.

The exclusion criteria for the group with OSCC: any history of chemotherapy, radiotherapy, and surgery (except biopsy).

### 2.3 How to intervene

First, all the patients were examined. If they met the criteria for entering the study, signed written consent was obtained, and they were entered into the study. Saliva was collected from people according to the protocol of Navazesh (1993). According to the provided guidance, people were forbidden to smoke and drink alcohol 24 h before saliva collection and the day before saliva collection. They also rinsed their mouths with clean water before going to sleep (saliva collection is usually done in the morning). One hour before saliva collection, people were careful not to eat, drink, or brush their teeth. The study participants rinsed their mouths with normal saline before collecting saliva and swallowed the first secreted saliva immediately after the rinse. The process of sampling was done by stimulating mouthwatering without chewing. Approximately 5 mL of saliva was placed in clean, dry polyethylene bottles and immediately stored at −80°; to be subjected to immunological analysis by the enzyme‐linked immunosorbent assay (ELISA) method at the right time.

### 2.4 Study variables

**Figure 1:**
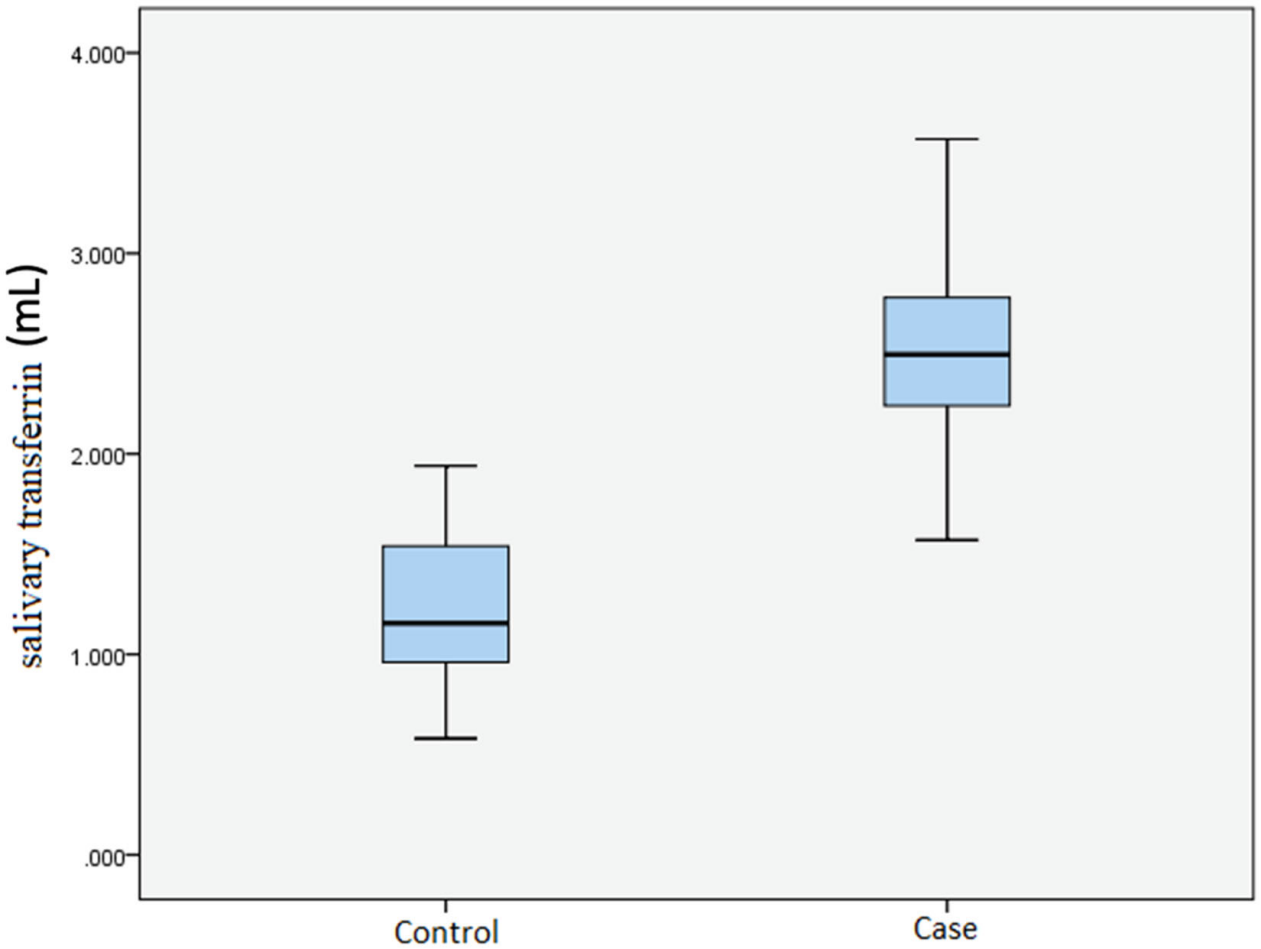
Boxplot diagram: Salivary transferrin variable in two groups under investigation.

Salivary transferrin is the quantitative dependent variable (Figure 1) of our study which was evaluated by the ELISA test.

The variables are mentioned in Table 1.

**Table 1:** Variables.

Control/evaluation method	Variable type			Scale type				Variables
				Qualitative		Quantitative		
	Confounding	Dependent	Independent	Ratings	Nominal	Relative	Interval	
ELISA test	□	■	□	□	□	■	□	Salivary transferrin
Check list	□	□	■	□	■	□	□	Study groups

Abbreviation: ELISA, enzyme‐linked immunosorbent assay.

### 2.5 Statistical analysis method

The data obtained from the study through descriptive statistical methods (prevalence‐percentage, mean ± standard deviation) and independent sample T‐test or its nonparametric equivalent Mann–Whitney *U* test. Data were analyzed using SPSS 17 statistical software. In this study, a *p*‐value of less than .05 was considered statistically significant.

## 3 RESULTS

The frequency and percentage of people participating in the study in two groups were 17 (42.5%) females and 23 (57.5%) males in the control group and 8 (20.0%) females and 32 (80.0%) males in the case group. Fisher's exact test was used to investigate the relationship between the gender of the participants in the study and the study groups. The significance level of the test was 0.050. The results of this test showed that there is no statistically significant relationship between the gender of the participants in the study and the study groups (*p*‐value = .053).

The mean and standard deviation of the age variable in the two groups under investigation were 57.85, 12.08 in the control group, and 62.12, 7.91 in the case group.

To check the existence of a statistically significant difference between the average age of the participants in the study, the independent samples T‐test was used in the two groups under investigation. The significance level of the test was 0.05. The results of this test (*p*‐value = .06) showed that there is no statistically significant difference between the average age of the participants in the study in the two groups under investigation (*p*‐value > .05). Among the patients participating in the study, the involvement site of 20 people (50%) was the lower lip, 7 (17.5%) soft palate, and 13 (32.5%) tongue.

The mean and standard deviation of the salivary transferrin variable in the control group were 1.23 mL and 0.37, respectively. While in the case group, the mean was 2.51 mL, and the standard deviation was 0.46.

To check the normality of the variable distribution of salivary transferrin, the Kolmogorov–Smirnoff test was used. The significance level of the test was .05. The results of this test showed that the variable distribution of salivary transferrin is normal. Therefore, a parametric test should be used to check the hypothesis of the study (*p*‐value > .05).

To check the existence of a statistically significant difference in the mean variable for salivary transferrin in the two study groups, the independent samples T‐Test was used. The significance level of the test was .05. The results of this test are given in Table 2.

**Table 2:** Independent samples T test results.

*p*‐Value	Degree of freedom	T statistic	Levene's test results	
			*p*‐Value	*F* statistic
**<.001**	**76**	**−13.43**	**.42**	**.63**

The results of the Independent Samples T‐Test were that the result of Levene's test with a *p*‐value equal to .42, the value of the *F* statistic was equal to .62, and the value of the T statistic with 76 degrees of freedom and *p*‐value < .001 equal to −13.43 was obtained. The results displayed that there is a statistically substantial difference between the mean of the salivary transferrin variable in the two study groups, and the mean of the salivary transferrin variable in the case group exceeds the mean in the control group (*p*‐value < .001).

## 4 DISCUSSION

Cancer is one of the most important causes of death and disability in the modern world. Cancers of the oral cavity comprise 3%–5% of all malignancies, and their incidence is increasing (Johnson et al., 2020).

SCC is the most common head and neck malignancy. OSCC, compared with skin squamous cell carcinoma (CSCC), has attracted special attention in studies due to its higher malignant potential (Shrestha et al., 2020). The common feature of all malignancies, as well as OSCC, is the identification and differential diagnosis of the disease in its advanced stages when the cancer has spread and made the patient's condition difficult (Farooq & Bugshan, 2020). Even with complex and advanced treatments, the prognosis is very low. Also, in most cases, the result is often not as expected, and it is accompanied by a lot of cost for the patient and the treatment system.

The early identification and diagnosis of cancer help in correct management, correct choice of treatment method, and effectiveness of the treatment. For this reason, researchers are always looking for effective and high‐efficiency methods for early detection of cancer. Biomarkers are one of these efforts to detect the disease early (Masaoutis et al., 2018). Transferrin, as a biomarker, is a protein that transports iron and can be measured directly. This protein is responsible for transporting iron between its production, consumption, and storage sites. Transferrin is essential for rapidly growing cells to grow (Silva et al., 2021). Cancerous cells develop rapidly. They are also implicated in the metabolic processes that require iron, such as DNA synthesis, metabolic signaling pathways, proliferation, electron transport, and cell survival (Banik et al., 2021; Buzalaf et al., 2020; Montaño‐Samaniego et al., 2020; Nakamura et al., 2021).

As in our study which the results showed an increase in transferrin in the study group of patients, recent studies have shown that transferrin improves transfection of cationic liposome/DNA complex into SCC cells (Faneca et al., 2019; Liu et al., 2020; Luiz et al., 2022). In a study, Jou et al. showed that salivary biomarkers, such as transferrin, have excellent susceptibility and specification for early OSCC diagnosis (Jou et al., 2010).

Markopoulos et al. investigated new molecular markers (DNA, RNA, and protein markers) from saliva and concluded that these markers can be used for diagnosis (Markopoulos et al., 2010). Shah et al. (2011) also reviewed salivary genomic and proteomic markers and observed that salivary biomarkers can be used for early diagnosis of malignancy. In a study conducted by Kinoshita et al. (2015) between 1996 and 2011 on 629 people with SCC, the level of tumor markers was measured in patients with the first stage, and a significant increase in these markers was observed. Moreover, in another study that Chen and his colleagues conducted on 534 patients between 2001 and 2013, the relationship between SCC antigens and tumor biomarkers after and before treatment was measured, and they concluded that this relationship can be used to diagnose malignancy (Chen et al., 2014).

Nagai et al. (2014) introduced a complex antibody against transferrin C receptor as a new biomarker for oral dysplasia and oral cancer. This monoclonal antibody was derived from a human genomic library by phage display method.

Although recent studies support the potential role of salivary transferrin for the diagnosis of oral cancer, it is not enough to be satisfied with a few limited studies. Accurate conclusions require more studies. Therefore, the present study was designed and implemented to evaluate the amount of salivary transferrin in patients with OSCC. In this study, 40 people with OSCC and 40 healthy people were selected based on the inclusion and exclusion criteria. After obtaining informed consent, they entered the study. With immunological analysis by ELISA method, the amount of salivary transferrin was calculated in the samples taken from the control and case groups. Moreover, the data obtained from the study were analyzed by statistical methods.

The results of the current study showed that there is a statistically significant difference between the mean of the salivary transferrin variable in the two groups under investigation, and the mean of the salivary transferrin variable in the case group is higher than its mean in the control group which is consistent with the results of previous studies.

Since the quick and timely diagnosis of diseases plays a major role in the timely and successful treatment of diseases, access to noninvasive sampling methods, such as saliva, is considered a desirable goal in examining diseases and their treatment results. The use of new diagnostic tools in the analysis of salivary components has multiplied the importance of saliva as a diagnostic tool. So the study on saliva becomes a suitable field for expanding the communication between the field of operations of dentists, who are mostly in contact with saliva, and doctors, who can use it to diagnose other systemic diseases. In general, more attention has been paid to saliva analysis and its use in disease diagnosis and public health monitoring. Considering the descriptive nature of the study and the existing discrepancies, there is a need for more studies to reach definite and analytical results.

There is a statistically significant difference between the variable means of salivary transferrin in the two groups under investigation. The mean variable of salivary transferrin in people with oral squamous cell malignancy is higher than the mean in healthy people.

## AUTHOR CONTRIBUTIONS

Mohammad Ali Ghavimi and Hosein Eslami did the conception and design. Dorna Abdolzadeh and Vahid Fakhrzadeh did the analysis, and interpretation of the data. Fatemeh Tavakoli and Aylar Afshari drafted the article and did the critical revision of the article for important intellectual content.

## CONFLICT OF INTEREST STATEMENT

The authors declare no conflict of interest.

## ETHICS STATEMENT

The study has been approved by the Tabriz University of Medical Science ethical committee. by ethical code IR.TBZMED.REC.1397.814. Written informed consent was obtained from the patient to publish this report in accordance with the journal's patient consent policy.

## Data Availability

None declared.

## References

[cre2809-bib-0001] Abati, S. , Bramati, C. , Bondi, S. , Lissoni, A. , & Trimarchi, M. (2020). Oral cancer and precancer: A narrative review on the relevance of early diagnosis. International Journal of Environmental Research and Public Health, 17(24):9160. https://www.mdpi.com/1660-4601/17/24/9160/htm 33302498 10.3390/ijerph17249160 PMC7764090

[cre2809-bib-0002] Al‐Garadi, M. A. , Mohamed, A. , Al‐Ali, A. K. , Du, X. , Ali, I. , & Guizani, M. (2020). A survey of machine and deep learning methods for Internet of Thing (IoT) security. IEEE Communications Surveys & Tutorials, 22(3), 1646–1685.

[cre2809-bib-0003] Andrade, R. G. D. , Veloso, S. R. S. , & Castanheira, E. M. S. (2020). Shape anisotropic iron oxide‐based magnetic nanoparticles: Synthesis and biomedical applications. International Journal of Molecular Sciences, 21(7), 2455. https://www.mdpi.com/1422-0067/21/7/2455/htm 32244817 10.3390/ijms21072455 PMC7178053

[cre2809-bib-0004] Banik, S. , Melanthota, S. K. , Arbaaz, A. , Vaz, J. M. , Kadambalithaya, V. M. , Hussain, I. , Dutta, S. , & Mazumder, N. (2021). Recent trends in smartphone‐based detection for biomedical applications: a review. Analytical and Bioanalytical Chemistry, 413(9), 2389–2406. 10.1007/s00216-021-03184-z 33586007 PMC7882471

[cre2809-bib-0005] Buzalaf, M. A. R. , Ortiz, A. C. , Carvalho, T. S. , Fideles, S. , Araújo, T. T. , Moraes, S. M. , Buzalaf, N. R. , & Reis, F. N. (2020). Saliva as a diagnostic tool for dental caries, periodontal disease and cancer: Is there a need for more biomarkers? Expert Review of Molecular Diagnostics, 20(5), 543–555. 10.1080/14737159.2020.1743686 32223655

[cre2809-bib-0006] Chadha, U. , Bhardwaj, P. , Agarwal, R. , Rawat, P. , Agarwal, R. , Gupta, I. , Panjwani, M. , Singh, S. , Ahuja, C. , Selvaraj, S. K. , Banavoth, M. , Sonar, P. , Badoni, B. , & Chakravorty, A. (2022). Recent progress and growth in biosensors technology: A critical review. Journal of Industrial and Engineering Chemistry, 109, 21–51.

[cre2809-bib-0007] Chen, H. , Liu, X. , Jin, Z. , Gou, C. , Liang, M. , Cui, L. , & Zhao, X. (2018). A three miRNAs signature for predicting the transformation of oral leukoplakia to oral squamous cell carcinoma. American Journal of Cancer Research, 8(8), 1403–1413. 30210912 PMC6129488

[cre2809-bib-0008] Chen, I. H. , Liao, C. T. , Wang, H. M. , Huang, J. J. , Kang, C. J. , & Huang, S. F. (2014). Using SCC antigen and CRP levels as prognostic biomarkers in recurrent oral cavity squamous cell carcinoma. PLoS One, 9(7), e103265. 10.1371/journal.pone.0103265 25061977 PMC4111511

[cre2809-bib-0009] De Cicco, D. , Tartaro, G. , Ciardiello, F. , Fasano, M. , Rauso, R. , Fiore, F. , Spuntarelli, C. , Troiano, A. , Lo Giudice, G. , & Colella, G. (2021). Health‐related quality of life in oral cancer patients: Scoping review and critical appraisal of investigated determinants. Cancers, 13(17), 4398. https://www.mdpi.com/2072-6694/13/17/4398/htm 34503208 10.3390/cancers13174398 PMC8431462

[cre2809-bib-0010] D'Souza, W. , & Kumar, A. (2020). microRNAs in oral cancer: Moving from bench to bed as next generation medicine. Oral Oncology, 111, 104916. 32711289 10.1016/j.oraloncology.2020.104916

[cre2809-bib-0011] Eslami, H. , Jamali, Z. , Babaei, H. , Fakhrzadeh, V. , Ahamdi, J. , & Tavakoli, F. (2022). Evaluation of the effect of grape seed extract (GSE) on oral mucositis in patients with head and neck radiotherapy history: A randomized clinical trial. International Journal of Cancer Management, 15(11), 130603. https://brieflands.com/articles/ijcm-130603.html

[cre2809-bib-0012] Faneca, H. , Düzgüneş, N. , & Pedroso de Lima, M. C. (2019). Suicide gene therapy for oral squamous cell carcinoma. In N. Düzgüneş (Ed.), Methods in molecular biology (Vol. 1895, pp. 43–55). Humana Press. https://link.springer.com/protocol/10.1007/978-1-4939-8922-5_4 30539528 10.1007/978-1-4939-8922-5_4

[cre2809-bib-0013] Farhadian, S. , Hashemi‐Shahraki, F. , Amirifar, S. , Asadpour, S. , Shareghi, B. , Heidari, E. , Shakerian, B. , Rafatifard, M. , & Firooz, A. R. (2022). Malachite green, the hazardous materials that can bind to Apo‐transferrin and change the iron transfer. International Journal of Biological Macromolecules, 194, 790–799. 34838577 10.1016/j.ijbiomac.2021.11.126

[cre2809-bib-0014] Farooq, I. , & Bugshan, A. (2020). Oral squamous cell carcinoma: metastasis, potentially associated malignant disorders, etiology and recent advancements in diagnosis. F1000Research, 9, 229. 32399208 10.12688/f1000research.22941.1 PMC7194458

[cre2809-bib-0015] Halib, N. , Rahman, N. Z. A. , Hanafiah, R. M. , Roslan, N. , & Jauhar, N. (2019). A simplified system for simulation of *Streptococcus mutans* biofilm on healthy extracted human tooth as dental plaque model. Journal of Applied Pharmaceutical Science, 9(2), 112–115. https://japsonline.com/abstract.php?article_id=2848&sts=2

[cre2809-bib-0016] Huang, Z. , Yang, X. , Huang, Y. , Tang, Z. , Chen, Y. , Liu, H. , Huang, M. , Qing, L. , Li, L. , Wang, Q. , Jie, Z. , Jin, X. , & Jia, B. (2023). Saliva ‐ A new opportunity for fluid biopsy. Clinical Chemistry and Laboratory Medicine, 61(1), 4–32. 10.1515/cclm-2022-0793/html 36285724

[cre2809-bib-0017] Johnson, D. E. , Burtness, B. , Leemans, C. R. , Lui, V. W. Y. , Bauman, J. E. , & Grandis, J. R. (2020). Head and neck squamous cell carcinoma. Nature Reviews Disease Primers, 6(1), 1–22. https://www.nature.com/articles/s41572-020-00224-3 10.1038/s41572-020-00224-3 PMC7944998 33243986

[cre2809-bib-0018] Jou, Y. J. , Lin, C. D. , Lai, C. H. , Chen, C. H. , Kao, J. Y. , Chen, S. Y. , Tsai, M. H. , Huang, S. H. , & Lin, C. W. (2010). Proteomic identification of salivary transferrin as a biomarker for early detection of oral cancer. Analytica Chimica Acta, 681(1–2), 41–48. 21035601 10.1016/j.aca.2010.09.030

[cre2809-bib-0019] Kawabata, H. (2019). Transferrin and transferrin receptors update. Free Radical Biology and Medicine, 133, 46–54. 29969719 10.1016/j.freeradbiomed.2018.06.037

[cre2809-bib-0020] Kaya, S. I. , Ozcelikay, G. , Mollarasouli, F. , Bakirhan, N. K. , & Ozkan, S. A. (2022). Recent achievements and challenges on nanomaterial based electrochemical biosensors for the detection of colon and lung cancer biomarkers. Sensors and Actuators B: Chemical, 351, 130856.

[cre2809-bib-0021] Kinoshita, T. , Ohtsuka, T. , Yotsukura, M. , Asakura, K. , Goto, T. , Kamiyama, I. , Otake, S. , Tajima, A. , Emoto, K. , Hayashi, Y. , & Kohno, M. (2015). Prognostic impact of preoperative tumor marker levels and lymphovascular invasion in pathological stage I adenocarcinoma and squamous cell carcinoma of the lung. Journal of Thoracic Oncology, 10(4), 619–628. 25634009 10.1097/JTO.0000000000000480

[cre2809-bib-0022] Kwong, G. A. , Ghosh, S. , Gamboa, L. , Patriotis, C. , Srivastava, S. , & Bhatia, S. N. (2021). Synthetic biomarkers: A twenty‐first century path to early cancer detection. Nature Reviews Cancer, 21(10), 655–668. https://www.nature.com/articles/s41568-021-00389-3 34489588 10.1038/s41568-021-00389-3 PMC8791024

[cre2809-bib-0023] Liu, C. , Zhang, L. , Zhu, W. , Guo, R. , Sun, H. , Chen, X. , & Deng, N. (2020). Barriers and strategies of cationic liposomes for cancer gene therapy. Molecular Therapy ‐ Methods & Clinical Development, 18, 751–764. http://www.cell.com/article/S2329050120301649/fulltext 32913882 10.1016/j.omtm.2020.07.015 PMC7452052

[cre2809-bib-0024] Luiz, M. T. , Dutra, J. A. P. , Tofani, L. B. , de Araújo, J. T. C. , Di Filippo, L. D. , Marchetti, J. M. , Chorilli, M. (2022). Targeted liposomes: A nonviral gene delivery system for cancer therapy. Pharmaceutics, 14(4), 821. https://www.mdpi.com/1999-4923/14/4/821/htm 35456655 10.3390/pharmaceutics14040821 PMC9030342

[cre2809-bib-0025] Maia, L. L. , Peterle, G. T. , Dos Santos, M. , Trivilin, L. O. , Mendes, S. O. , De Oliveira, M. M. , dos Santos, J. G. , Stur, E. , Agostini, L. P. , Couto, C. V. M. S. , Dalbó, J. , de Assis, A. L. E. M. , Archanjo, A. B. , Mercante, A. M. D. C. , Lopez, R. V. M. , Nunes, F. D. , de Carvalho, M. B. , Tajara, E. H. , Louro, I. D. , & Álvares‐da‐Silva, A. M. (2018). JMJD1A, H3K9me1, H3K9me2 and ADM expression as prognostic markers in oral and oropharyngeal squamous cell carcinoma. PLoS One, 13(3), e0194884. 10.1371/journal.pone.0194884 29590186 PMC5874045

[cre2809-bib-0026] Markopoulos, A. K. , Michailidou, E. Z. , & Tzimagiorgis, G. (2010). Salivary markers for oral cancer detection. The Open Dentistry Journal, 4(1), 172–178. 21673842 10.2174/1874210601004010172 PMC3111739

[cre2809-bib-0027] Masaoutis, C. , Mihailidou, C. , Tsourouflis, G. , & Theocharis, S. (2018). Exosomes in lung cancer diagnosis and treatment. From the translating research into future clinical practice. Biochimie, 151, 27–36. 29857182 10.1016/j.biochi.2018.05.014

[cre2809-bib-0028] Montaño‐Samaniego, M. , Bravo‐Estupiñan, D. M. , Méndez‐Guerrero, O. , Alarcón‐Hernández, E. , & Ibáñez‐Hernández, M. (2020). Strategies for targeting gene therapy in cancer cells with tumor‐specific promoters. Frontiers in Oncology, 10, 605380. 33381459 10.3389/fonc.2020.605380 PMC7768042

[cre2809-bib-0029] Mortazavi, H. , Safi, Y. , Baharvand, M. , Jafari, S. , Anbari, F. , & Rahmani, S. (2019). Oral white lesions: An updated clinical diagnostic decision tree. Dentistry Journal, 7(1), 15. https://www.mdpi.com/2304-6767/7/1/15/htm 30736423 10.3390/dj7010015 PMC6473409

[cre2809-bib-0030] Mountcastle, S. E. , Cox, S. C. , Sammons, R. L. , Jabbari, S. , Shelton, R. M. , & Kuehne, S. A. (2020). A review of co‐culture models to study the oral microenvironment and disease. Journal of Oral Microbiology, 12(1):1773122. 10.1080/2000229720201773122 32922679 PMC7448840

[cre2809-bib-0031] Nagai, K. , Nakahata, S. , Shimosaki, S. , Tamura, T. , Kondo, Y. , Baba, T. , Taki, T. , Taniwaki, M. , Kurosawa, G. , Sudo, Y. , Okada, S. , Sakoda, S. , & Morishita, K. (2014). Development of a complete human anti‐human transferrin receptor C antibody as a novel marker of oral dysplasia and oral cancer. Cancer Medicine, 3(4), 1085–1099. 10.1002/cam4.267 24890018 PMC4303177

[cre2809-bib-0032] Nakamura, K. , Hiyake, N. , Hamada, T. , Yokoyama, S. , Mori, K. , Yamashiro, K. , Beppu, M. , Sagara, Y. , Sagara, Y. , & Sugiura, T. (2021). Circulating microRNA panel as a potential novel biomarker for oral squamous cell carcinoma diagnosis. Cancers, 13(3), 449. https://www.mdpi.com/2072-6694/13/3/449/htm 33504017 10.3390/cancers13030449 PMC7865311

[cre2809-bib-0033] Narayanasamy Rajavelu, T. , Abimannane, A. , Chinnaiah Govindhareddy, D. K. , Kayal, S. , & Kar, R. (2020). Langerhans' cell histiocytosis masquerading as Caroli's disease. Journal of Pediatric Hematology/Oncology, 42(7), e620–e622. 31033792 10.1097/MPH.0000000000001495

[cre2809-bib-0034] Navazesh, M. (1993). Methods for collecting saliva. Annals of the New York Academy of Sciences, 694(1), 72–77. 10.1111/j.1749-6632.1993.tb18343.x 8215087

[cre2809-bib-0035] Piemonte, E. D. , Lazos, J. P. , Gilligan, G. M. , Panico, R. L. , Werner, L. C. , Yang, Y. H. , & Warnakulasuriya, S. (2022). Chronic mechanical irritation enhances the effect of tobacco and alcohol on the risk of oral squamous cell carcinoma: A case‐control study in Argentina. Clinical Oral Investigations, 26(10), 6317–6326. 10.1007/s00784-022-04584-w 35727376

[cre2809-bib-0036] Riccardi, G. , Bellizzi, M. G. , Fatuzzo, I. , Zoccali, F. , Cavalcanti, L. , Greco, A. , Vincentiis, M. , Ralli, M. , Fiore, M. , Petrella, C. , Minni, A. , & Barbato, C. (2022). Salivary biomarkers in oral squamous cell carcinoma: A proteomic overview. Proteomes, 10(4), 37. https://www.mdpi.com/2227-7382/10/4/37/htm 36412636 10.3390/proteomes10040037 PMC9680331

[cre2809-bib-0037] Roi, A. , Roi, C. I. , Negruțiu, M. L. , Riviș, M. , Sinescu, C. , & Rusu, L. C. (2020). The challenges of OSCC diagnosis: Salivary cytokines as potential biomarkers. Journal of Clinical Medicine, 9(9), 2866. https://www.mdpi.com/2077-0383/9/9/2866/htm 32899735 10.3390/jcm9092866 PMC7565402

[cre2809-bib-0038] Shah, F. D. , Begum, R. , Vajaria, B. N. , Patel, K. R. , Patel, J. B. , Shukla, S. N. , & Patel, P. S. (2011). A review on salivary genomics and proteomics biomarkers in oral cancer. Indian Journal of Clinical Biochemistry, 26(4), 326–334. 10.1007/s12291-011-0149-8 23024467 PMC3210231

[cre2809-bib-0039] Shen, Y. , Li, X. , Dong, D. , Zhang, B. , Xue, Y. , & Shang, P. (2018). Transferrin receptor 1 in cancer: A new sight for cancer therapy. American Journal of Cancer Research, 8(6), 916. 30034931 PMC6048407

[cre2809-bib-0040] Shrestha, A. D. , Vedsted, P. , Kallestrup, P. , & Neupane, D. (2020). Prevalence and incidence of oral cancer in low‐ and middle‐income countries: A scoping review. European Journal of Cancer Care, 29(2), e13207. 10.1111/ecc.13207 31820851

[cre2809-bib-0041] Silva, A. M. N. , Moniz, T. , de Castro, B. , & Rangel, M. (2021). Human transferrin: An inorganic biochemistry perspective. Coordination Chemistry Reviews, 449, 214186.

[cre2809-bib-0042] Stepan, K. O. , Mazul, A. L. , Larson, J. , Shah, P. , Jackson, R. S. , Pipkorn, P. , Kang, S. Y. , & Puram, S. V. (2023). Changing epidemiology of oral cavity cancer in the United States. Otolaryngology–Head and Neck Surgery, 168(4), 761–768. 10.1177/01945998221098011 35503657 PMC10154079

[cre2809-bib-0043] Tenchov, R. , Sasso, J. M. , Wang, X. , Liaw, W. S. , Chen, C. A. , & Zhou, Q. A. (2022). Exosomes nature's lipid nanoparticles, a rising star in drug delivery and diagnostics. ACS Nano, 16(11), 17802–17846. 10.1021/acsnano.2c08774 36354238 PMC9706680

[cre2809-bib-0044] Tuominen, H. , & Rautava, J. (2021). Oral microbiota and cancer development. Pathobiology, 88(2), 116–126. 10.1159/000510979 33176328

[cre2809-bib-0045] Uma Maheswari, T. N. , Nivedhitha, M. S. , & Ramani, P. (2020). Expression profile of salivary micro RNA‐21 and 31 in oral potentially malignant disorders. Brazilian Oral Research, 34, e002. https://www.scielo.br/j/bor/a/bW8Wy5GTQF8gRn3RsTmDVgj/?lang=en 32049107 10.1590/1807-3107bor-2020.vol34.0002

